# RNA-Binding Protein TAF15 Suppresses Toxicity in a Yeast Model of FUS Proteinopathy

**DOI:** 10.3390/jof12050341

**Published:** 2026-05-06

**Authors:** Elliott Hayden, Aicha Kebe, Shuzhen Chen, Abagail Chumley, Chenyi Xia, Widad El-Zein, Quan Zhong, Shulin Ju

**Affiliations:** 1 Department of Biological Sciences, Wright State University, Dayton, OH 45435, USA; 2 School of Basic Medicine, Shanghai University of Traditional Medicine, Shanghai 201203, China

**Keywords:** ALS, FET proteins, genetic screen, p-bodies, stress granules

## Abstract

Mutations in an RNA-binding protein FUS are known to cause familial amyotrophic lateral sclerosis (ALS). Since this discovery, mutations in several other RNA-binding proteins (RBPs) have also been linked to ALS. Some of these ALS-associated RBPs have been shown to colocalize with ribonucleoprotein (RNP) granules such as stress granules and processing bodies (p-bodies). Increasing evidence has emerged supporting a hypothesis that the impaired clearance, inappropriate assembly, and dysregulation of RNP granules play a role in ALS. Through the genome-scale overexpression screening of a yeast model of FUS toxicity, we found that TAF15, a human RBP with a similar protein domain structure and belonging to the same FET protein family as FUS, suppresses FUS toxicity in yeast. The suppression by TAF15 is specific to FUS and not found in other yeast models of neurodegenerative disease-associated proteins. We showed that the RNA recognition motif (RRM) of TAF15 is required for its suppression of FUS toxicity. Furthermore, FUS and TAF15 physically interact, and the C-terminus of TAF15 is required for both the physical protein–protein interaction and its protection against FUS toxicity. Finally, while FUS induces and colocalizes with both stress granules and p-bodies, TAF15 only induces and colocalizes with p-bodies. Importantly, the co-expression of FUS and TAF15 induces more p-bodies than individually expressing each gene alone, and FUS toxicity is exacerbated in yeast that is deficient in p-body formation. Overall, our findings suggest a role of increased p-body formation in the suppression of FUS toxicity by TAF15.

## 1. Introduction

Amyotrophic lateral sclerosis (ALS) is a neurodegenerative disease characterized by the progressive degeneration of motor neurons resulting in paralysis and ultimately death within 2–5 years of symptom onset [1]. The precise mechanisms leading to motor neuron death are not known; however, RNA dysregulation has emerged as a prominent mechanism contributing to ALS pathogenesis [2,3].

A pathologic hallmark of ALS is the presence of cytoplasmic protein inclusions within affected neuronal cells. In the vast majority of cases, these inclusions contain the RNA-binding protein TAR DNA binding protein (TDP-43) [4,5]. Mutations in the gene encoding TDP-43 cause familial ALS and rare sporadic cases of ALS [6]. Like TDP-43, another RNA-binding protein, FUS, is mutated in ALS and accumulates in pathologic cytoplasmic inclusions in ALS patient tissue [7,8]. Over the last decade, mutations in several other genes encoding RBPs have been found in ALS patients, including hnRNPA1, hnRNPA2B1 [9], Matrin3 [10], TAF15 [11,12] and EWSR1 [13]. Proposed mechanisms leading to neurodegeneration in ALS include the mis-regulation of RNA metabolism through the sequestration of RNA and RBPs in pathologic inclusions, splicing dysregulation, RNA transport and stability defects, and the disruption of nonsense-mediated decay.

Yeast models have been widely used to study ALS-linked protein toxicity. It has been established that, when overexpressed in yeast cells, both TDP-43 and FUS form cytoplasmic inclusions and induce cytotoxicity [14,15,16]. Genetic screening using yeast models of neurodegenerative disease has been instrumental in identifying mechanisms of toxicity contributing to various neurological disorders as well as developing candidate therapeutic targets [15,16,17,18,19,20,21]. This screening pipeline relies on screening against a library of 5500 yeast genes and then identifying and testing the human homologs of the yeast suppressors for their effects on FUS toxicity [22]. Our lab and others have taken advantage of the yeast model system to perform genome-wide screening to identify modifiers of ALS protein toxicity. We identified that a yeast gene encoding an RNA-binding protein, Ecm32, protects yeast cells against FUS-induced toxicity [16]. Its human homolog, hUPF1, is an ATP-dependent RNA helicase associated with stress granules, and is a key regulator of the nonsense-mediated mRNA decay pathway [23]. Subsequently, we found that the expression of hUPF1 is protective against ALS protein toxicity in mammalian neurons as well as in a rat model of TDP-43 [24,25]. This supports the hypothesis that there are conserved mechanisms underlying the rescue of FUS toxicity in yeast and mammalian cells.

Importantly, the expression of hUPF1 in yeast protects cells from the toxicity induced by FUS and TDP-43 [16]. These findings motivated the study that human-gene modifiers can be directly identified in yeast models. The direct screening of human genes would provide greater coverage and allow the identification of suppressors without obvious yeast homologs [26]. We identified 37 human genes that, when overexpressed, strongly suppress FUS toxicity [26]. The identified suppressor genes are enriched in genes encoding RNA-binding proteins, including TAF15.

TAF15 is a member of the FET family of RPBs, which includes FUS and EWSR1. These proteins share similar structural and functional features and participate in the regulation of transcription, mRNA processing, and nucleocytoplasmic transport [27,28]. Like FUS, TAF15 accumulates into cytoplasmic stress granules under stress conditions [29,30]. All three FET proteins are present in the FUS-positive cytoplasmic inclusions found in patients with ALS and Frontotemporal Lobar Degeneration (FTLD) [11,12,29,31].

In this study, we characterized the suppressor effect of TAF15 on FUS toxicity to better understand the mechanism of FUS toxicity. We found that TAF15 specifically suppressed the toxicity of FUS, while having no effect on cellular toxicity induced by a few other proteinopathies previously established in yeast. While TAF15 does not change the cytoplasmic localization or protein level of FUS, the RNA recognition motif (RRM) in TAF15 is essential for its suppression effect. We observed a physical protein–protein interaction between FUS and TAF15 that is also required for the suppression of FUS toxicity. We show that TAF15 induces and localizes to p-bodies in yeast. Unlike FUS, TAF15 does not induce or associate with stress granules. When FUS and TAF15 are co-expressed, the induction of p-bodies is stronger than the individual expression of each protein. Moreover, FUS toxicity is exacerbated in mutant yeast strains that are deficient in the formation of p-bodies. Taken together, our data suggest a role of p-body induction in the suppression of FUS toxicity by TAF15.

## 2. Materials and Methods

### 2.1. Yeast Strains, Media and Plasmids

The 1XFUS integration strain was generated in the haploid W303 yeast strain (*MAT*a can1-100, leu2-3,112, trp1-1, ura3-1, ade2-1, his3-11,15::pRS303Gal1FUS), as previously described [16]. The FUS-YFP strain was generated by transforming pRS413Gal1FUS-YFP into the haploid W303 yeast strain (*MAT*a can1-100, leu2-3, 112, trp1-1, ura3-1, ade2-1, his3-11,15). Yeast transformed with free (non-integrating) plasmids were grown in synthetic complete media containing all amino acids except for those used for selection of the plasmid. Media contained either 2% dextrose, galactose or raffinose as the carbon source. Yeast was grown at 30 °C with shaking (200 rpm) for liquid cultures.

An LR reaction was used to transfer genes from gateway entry clones to gateway-compatible yeast destination vectors [32]. Products of the LR reaction were transformed into DH5α-competent bacteria using standard bacterial transformation methods. Expression clones were extracted and confirmed with restriction enzyme digestion and sequencing.

To generate a pRS413Gal1FUS-YFP plasmid, an BP reaction was used to transfer a full-length FUS gene from pRS426Gal-FUS-YFP (Addgene, Boston, MA, USA) [15] onto gateway entry clone pDONR223FUS. FUS was then transferred to the gateway destination vector pRS413Gal1ccdB-YFP for making the yeast expression construct of C-terminal YFP-tagged FUS. The expression plasmid was confirmed by DNA sequencing.

To generate TAF15 RRM mutant plasmids (TAF15 Mut^RRM^), a DNA fragment containing TAF15’s RRM domain with four phenylalanine mutations (F254L, F290L, F308L, and F317L) was synthesized and cloned into the gateway entry vector pDONR223. The final product, pDONR223-TAF15 Mut^RRM^, was confirmed by sequencing. A gateway LR reaction was then used to shuttle TAF15 Mut^RRM^ into various destination vectors. EWSR1 was similarly cloned into gateway destination vectors.

Site-directed mutagenesis was used to generate the TAF15 N-terminal deletion mutant (TAF15ΔN) and C-terminal deletion mutant (TAF15ΔC). Mutagenesis was performed using the QuickChange II Site-Directed Mutagenesis Kit (Agilent, Santa Clara, CA, USA) according to the manufacturer’s instructions. Mutagenic primers were designed online using the QuickChange Primer Design Program. Mutations were verified by DNA sequencing.

### 2.2. Yeast Transformation

Yeast expression constructs were transformed using the standard PEG/lithium acetate method. Cells were then spread onto their respective synthetic amino acid dropout plates selecting for the presence of the plasmid and grown at 30 °C for 3 to 4 days.

### 2.3. Serial Dilution Growth Assays

Yeast cultures were grown overnight in synthetic media containing raffinose as the carbon source. Cultures were normalized to an OD_600_ = 1.0, 5× serially diluted, and spotted to their respective synthetic agar plates containing 2% glucose to shut down gene expression (Gene Off) or galactose (Gene On) to induce gene expression. Agar plates were incubated at 30 °C for 4 days, and pictures of the plates were taken every 24 h.

### 2.4. Yeast Two-Hybrid (Y2H) Assay

AD Y2H constructs were transformed into the yeast strain Y8800 (*MAT*a leu2-3,112 trp1-901 his3Δ200 ura3-52 gal4Δ gal80Δ GAL2::ADE2 GAL1::HIS3@LYS2 GAL7::lacZ@met2 cyh2R) and DB Y2H constructs were transformed into the yeast strain Y8930 (*MAT*α leu2-3,112 trp1-901 his3Δ200 ura3-52 gal4Δ gal80Δ GAL2::ADE2 GAL1::HIS3@LYS2 GAL7::lacZ@met2 cyh2R). DB and AD yeast strains of the opposite mating types were crossed on a YPD agar plate. Diploid yeast from YPD plates were selected on SD/-Leu/-Trp agar plates. For the Y2H interaction assay, 3 μL of diploid yeast growing in SD/-Leu/-Trp media were spotted on SD/-Leu/-Trp (as control) and selective SD/-Leu/-Trp/-His plates. Growth in the absence of histidine indicates a positive protein–protein interaction [33]. Both the control and selective plates were incubated at 30 °C for 5 days and pictures of the plates were taken every 24 h.

### 2.5. Western Blotting

Yeast crude extract was prepared using a post-alkaline extraction method. Protein was subjected to SDS/PAGE and transferred to a PVDF membrane (Millipore, Burlington, MA, USA). The membrane was rinsed with water and then blocked with 5% nonfat dry milk in TBST for 1 h. The membrane was then incubated with the primary antibody overnight at 4 °C. After being washed, the membrane was incubated with an alkaline phosphatase-conjugated secondary antibody for 1 h. The membrane was washed again in TBST and then developed with one-step NBT/BCIP solution (Thermo fisher, Cincinnati, OH, USA). The anti-FUS (Abcam, Boston, MA, USA), anti-PGK1 (Invitrogen, Carlsbad, CA, USA), anti-TAF15 (Abcam, Boston, MA, USA) antibodies, AP-conjugated anti-rabbit and anti-mouse secondary antibodies were used at a dilution of 1:10,000. The image was taken using the epi-illumination colorimetric capture option of Amersham Imager 600 (GE healthcare technology, Chicago, IL, USA). Image quantification was performed using Amersham Imager 600 analysis workflow. Briefly, background was subtracted using a minimum profile method and protein bands were detected and PGK1 bands were specified as the reference bands.

### 2.6. Fluorescence Microscopy

Yeast strains were grown in synthetic media containing raffinose to the mid-log phase. Cultures were centrifuged, washed with sterile ddH_2_O twice, and then resuspended in media containing 2% galactose to induce protein expression for 6 h unless otherwise specified. Cultures were then harvested and placed on a microscope slide with a cover slip. Images were obtained with an Olympus IX83 inverted fluorescent microscope (Olympus, Center Valley, PA, USA) at either 40× or 100× (oil immersion) magnification using FITC, EYFP, ECFP and TxRed filter cubes. For multi-color imaging experiments, bleed-through experiments were first conducted with each fluorescently tagged protein of interest individually to make sure that signal from the fluorescently tagged protein was only visible in its appropriate filter. The YFP and brightfield images were merged in ImageJ (version 1.54g) and the cell counter plugin was used to count cells and FUS-YFP foci. First, without seeing the YFP signal, the total number of cells (300–500 cells per field) in a field of view were counted. Next, the number of cells in the field without any visible FUS foci were counted by analyzing the merged brightfield and YFP channels together. Data were collected from three independent results and statistical analysis was performed.

### 2.7. Quantification of P-Bodies

Yeast strains were grown overnight at 30 °C to the mid-log phase (OD_600_ = 0.5) in Raffinose-containing media. Cultures were induced with galactose for 6 h, as described above. Live yeast cells were imaged at 100× magnification using EYFP, ECFP and TxRed filter cubes. P-bodies in the TxRed channel were detected by the count and measure function of Olympus cellSens Dimension software (version 1.18). The number of yeast cells in the image was manually counted using the bright field image.

### 2.8. Quantitative Analysis of Yeast Spotting Assay

The analysis of yeast spotting was carried out as described in STAR protocols “A Quantitative Imaging-Based Protocol for Yeast Growth and Survival on Agar Plates.”. Images of the agar plates from 3 days of growth were imported into ImageJ and the background was subtracted using the sliding paraboloid option. The uniformity of the background gray value signal of the agar plate was confirmed by measuring the gray value signal from multiple places of the plate. Yeast growth was quantified by measuring the gray value signal of the yeast spots for all the strains for the second dilution (5×). The gray value of the background was subtracted from the gray value of the yeast spots and the resulting values were normalized to the value of the control strain. This was repeated for a total of three biological replicates with calculation of the mean and standard error.

### 2.9. Statistical Analysis

All results from statistical analysis are presented as mean  ±  standard error of the mean (SEM) and differences were considered significant when *p*  <  0.05. Significance is presented as ** *p*  <  0.01. For comparison of two groups, two-tailed unpaired Student’s *t*-test was used in combination with an F-test to confirm that the variances between groups were not significantly different. Data were analyzed by using the GraphPad Prism version 8.0.

## 3. Results

### 3.1. Expression of the RNA-Binding Protein TAF15 Rescues FUS Toxicity

**Figure 1:**
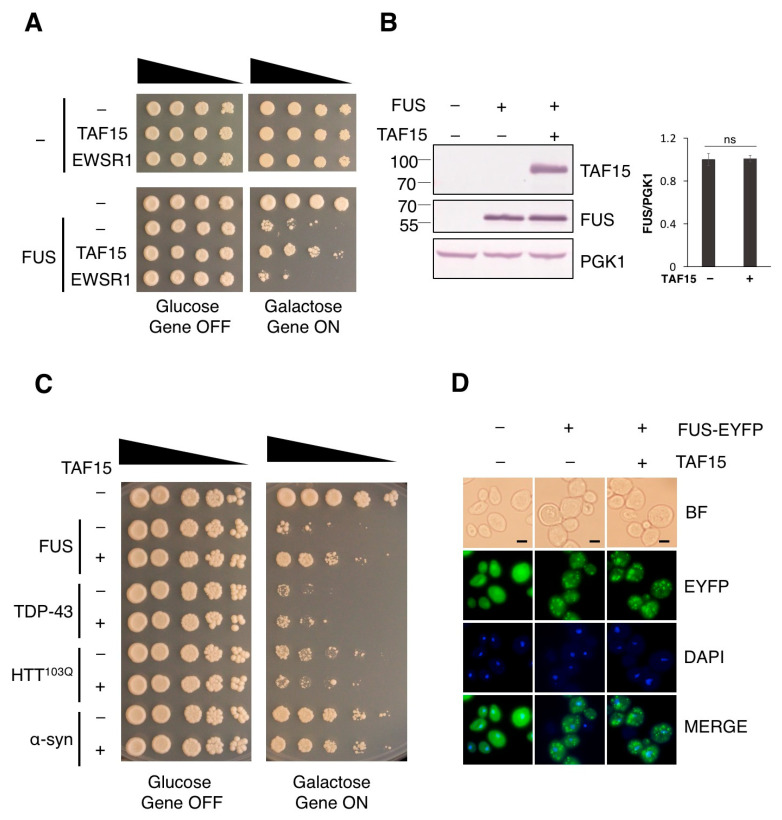
**TAF15 rescues FUS toxicity.** (**A**) Serial dilution growth assay was performed using the 1XFUS model transformed with the pAG416Gal-ccdB vector control (-), TAF15 or EWSR1 expression plasmid. Wild-type yeast strain transformed with the vector (Top-) was used as a control strain showing no toxicity. (**B**) Expression of FUS protein was examined in the 1XFUS model transformed with the vector control (-) or the TAF15 expression plasmid. Yeast containing empty vectors was used as a control strain without FUS or TAF15 expression. Protein expression was induced with 2% galactose for 6 h in the indicated strains. Western blot analysis was performed using an antibody against FUS, TAF15 and loading control PGK1. FUS protein levels were detected and normalized to the level of the PGK1, as indicated by the values on the right (ns, not statistically significant). (**C**) Serial dilution growth assay using yeast models of TDP-43, HTT103Q, and α-synuclein (IntTox) [14,33,35] transformed with the vector control (-) or the TAF15 expression plasmid (+). Wild-type yeast transformed with two empty vectors serves as a no-toxicity control. (**D**) Yeast expressing FUS-YFP and transformed with the vector control (-) or the TAF15 expression plasmid (+) were grown in media containing 2% galactose for 6 h to induce protein expression, then fixed and stained with DAPI. A yeast strain expressing YFP was used as a control. Imaging was performed at 100× magnification. Scale bar—2 μm. Images are representatives from three independent experiments.

To directly identify human suppressor genes of FUS-induced toxicity, we performed a genome-scale genetic screen using a library containing 13,570 human genes individually cloned in an inducible yeast expression vector [26]. Through this work, we identified TAF15 as a suppressor of FUS toxicity (Figure 1A). TAF15, FUS and EWSR1 are three members of the FET protein family. EWSR1 was not included in the human-gene library used for the screen. Given the functional and structural similarities between the three FET proteins, we also tested the effect of EWSR1 on FUS toxicity. We individually cloned EWSR1 in the same expression vector and performed serial dilution growth assays in the yeast model of FUS. Unlike TAF15, EWSR1, at similar expression level does not suppress the toxicity of FUS (Figure 1A).

It has been established that the toxicity of FUS is dose-dependent in yeast [16]. To test whether TAF15 is acting by decreasing FUS protein level, we performed Western blot using antibodies against TAF15 and FUS. Upon six hours of induction, FUS protein levels were not altered by the expression of TAF15 (Figure 1B). Additionally, we performed Western blot for FUS protein at earlier (3 h) and later time points (22 h) in TAF15 expression, and FUS protein levels remained unchanged by TAF15 (Supplementary Figure S1).

To investigate if the suppressor effect of TAF15 is specific to FUS-mediated protein toxicity, we expressed TAF15 in several other well-established yeast models of neurodegenerative disease proteotoxicity, including another ALS-linked protein, (TDP-43), a Huntington’s disease-associated protein (mutant huntingtin fragment HTT103Q) and a Parkinson’s disease-associated protein (α-synuclein) [14,18,34,35,36,37]. Toxicity caused by these proteins seems to occur largely through different mechanisms as genetic screening for modifiers of toxicity has uncovered different sets of modifier genes [15,18,20,21,38,39]. Serial dilution growth assays of each of these yeast models showed that TAF15 only protects cells against FUS toxicity, while having no effect on the toxicity caused by TDP-43, HTT103Q or α-synuclein (Figure 1C).

FUS contains a non-classical proline–tyrosine nuclear localization signal (PY-NLS) in its extreme C-terminus, which allows FUS to shuttle between the nucleus and cytoplasm. FUS nuclear localization signal is non-functional in yeast, resulting in the cytoplasmic localization and toxicity of FUS. Increasing the nuclear localization of FUS by adding a functional yeast NLS to the C-terminus of FUS suppressed its toxicity [16]. Promoting the nuclear localization of FUS has been shown as a mechanism to reduce toxicity in several other model systems as well [40,41,42]. We therefore questioned whether TAF15 restores FUS localization to the nucleus. To test this possibility, we generated a yeast strain expressing YFP-tagged FUS, which exhibits similar toxicity to the non-tagged FUS model (Figure S2). Upon the co-expression of TAF15, FUS remained localized to cytoplasmic foci (Figure 1D), indicating that TAF15 is not acting by promoting the nuclear localization of FUS.

### 3.2. RNA and Protein Interaction Domains of TAF15 Are Required for the Rescue of FUS Toxicity

**Figure 2:**
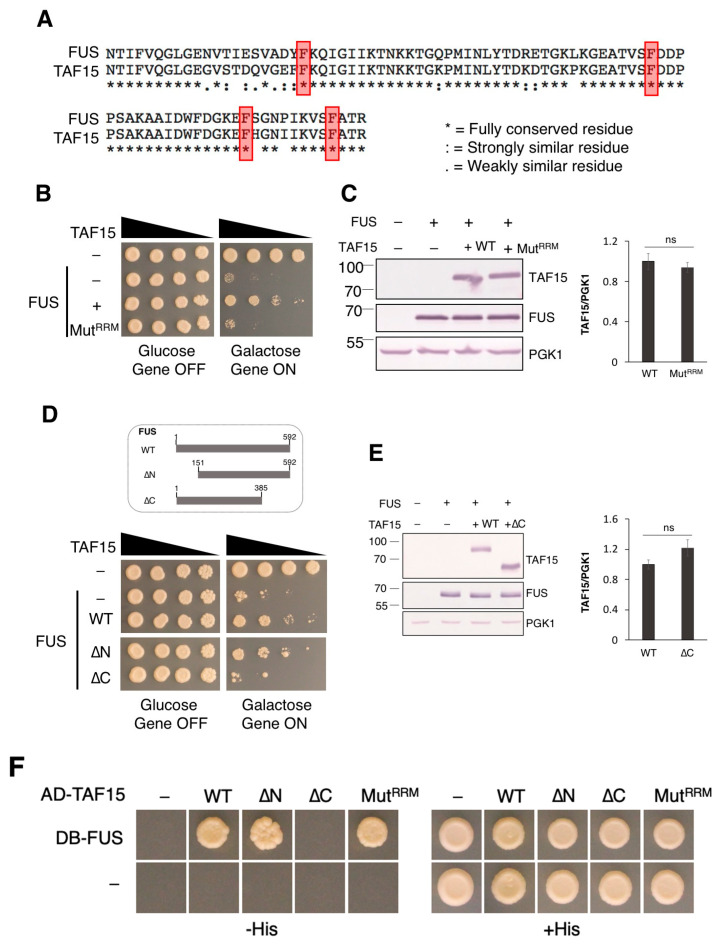
**RRM and the C-terminal interaction domain of TAF15 are required for rescuing FUS toxicity.** (**A**) Alignment of the RRM of FUS (amino acids 285–371) and TAF15 (amino acids 234–320). Conserved phenylalanine residues highlighted in red are important for RNA binding and toxicity of FUS [15,43,44]. (**B**) Serial dilution growth assay was performed using the 1XFUS model transformed with the vector control (-) or the expression plasmid containing wild-type TAF15 (+) or the RRM mutant of TAF15 (Mut^RRM^). (**C**) All strains used in (**B**) were induced with 2% galactose for 6 h and Western blot was performed using an antibody against FUS, TAF15 and the loading control protein PGK1. Shown on the right is the relative expression levels of wild-type and mutant TAF15 (Mut^RRM^) when FUS was overexpressed, quantitated from average of three independent experiments with the wild-type sample of TAF15 set to 1. (**D**) On the top is the schematic representation of the truncated TAF15 used in this experiment. On the bottom is the serial dilution growth assay that was performed using the 1XFUS model transformed with the vector control (-) or the expression plasmid containing wild-type TAF15 (+), the N-terminal deletion (ΔN) or the C-terminal deletion (ΔC) mutant. (**E**) All strains used in (**D**) were induced with 2% galactose for 6 h in the indicated strains. Western blot was performed. Shown on the right is the relative expression levels of wild-type and mutant TAF15 (ΔC) when FUS was overexpressed, quantitated from average of three independent experiments with the wild-type sample of TAF15 set to 1 (ns not statistically significant). (**F**) Y2H interaction assay was performed between FUS and the wild-type and mutant TAF15 proteins, including the N-terminal deletion (ΔN), the C-terminal deletion (ΔC) and the RRM mutant. Images from (**B**–**F**) are representatives of three independent experiments.

Both FUS and TAF15 contain several conserved protein domains and share a similar domain structure. One of their most highly conserved domains is their RNA recognition motif (RRM). The RRM of FUS and TAF15 RRM are 85% identical (Figure 2A), while the RRM of FUS and EWSR1 are less similar (60%). RRM alignment showed that TAF15 and FUS share the same conserved phenylalanine residues (Figure 2A, highlighted in red). Conserved phenylalanine residues in FUS RRM are responsible for its direct stacking interactions with RNA bases [43]. The mutation of conserved phenylalanine residues to leucine in FUS disrupt its binding to RNA, and render FUS non-toxic in yeast and drosophila models [15,44].

Given the similarity in RRMs between FUS and TAF15, we reasoned that these phenylalanine residues may be important for the RNA binding ability of TAF15 and its ability to rescue FUS toxicity. For example, TAF15 may compete for binding to a similar set of RNA targets in yeast as FUS. To test this possibility, we mutated four conserved phenylalanine residues within the RRM of TAF15 (Phe^254^, Phe^290^, Phe^308^ and Phe^317^) to leucine to produce a non-functional RRM mutant of TAF15 (Mut^RRM^). Unlike wild-type TAF15, Mut^RRM^ fails to rescue FUS toxicity (Figure 2B). Western blot demonstrated that the loss of rescue is not due to the abnormal expression of the mutant protein, as Mut^RRM^ was similarly expressed as wild-type TAF15 (Figure 2C). These data support our hypothesis that the rescue of FUS toxicity by TAF15 is dependent on its RRM.

It has previously been reported that the FET proteins interact with each other and are found in the same protein complex [45,46]. Using recombinant proteins in pull-down experiments, the N-terminal domains of FUS and TAF15 were found to form homo- and heterocomplexes with full-length versions of themselves, as well as with each other. In addition, a second binding region in the C-terminus of TAF15 was also identified [47]. We wondered if the physical interaction between FUS and TAF15 is required for the observed rescue of FUS toxicity. To test this possibility, we generated TAF15 mutants lacking its N terminus (TAF15ΔN, lacking amino acids 1–150) and a C-terminal region (TAF15ΔC, lacking amino acids 385–592). Next, we tested whether these TAF15 mutants retain their ability to suppress FUS toxicity. Interestingly, TAF15ΔN but not TAF15ΔC rescues FUS toxicity (Figure 2D). To confirm that the lack of rescue seen with TAF15ΔC is not due to abnormal expression of the mutant protein, Western blot was performed. TAF15ΔC was similarly expressed as the full-length wild-type protein (Figure 2E). Finally, to test whether FUS and TAF15 are interacting partners in our yeast model, we used the yeast two-hybrid (Y2H) assay, with FUS expressed as a DB (Gal4 DNA-binding domain) fusion protein and TAF15 expressed as an AD (Gal4 activation domain) fusion protein. Based on the ability of yeast to grow on media lacking the amino acid histidine due to the activation of the HIS3 reporter gene, wild-type TAF15 and FUS interact (Figure 2F). The same is true for TAF15ΔN and Mut^RRM^ mutants. The interaction between TAF15ΔC mutant and FUS is no longer detectable in the Y2H assay (Figure 2F). These findings support our hypothesis that the interaction between TAF15 and FUS is also required for TAF15 to act as a suppressor of FUS toxicity.

### 3.3. TAF15 Decreases Number of FUS Foci

**Figure 3:**
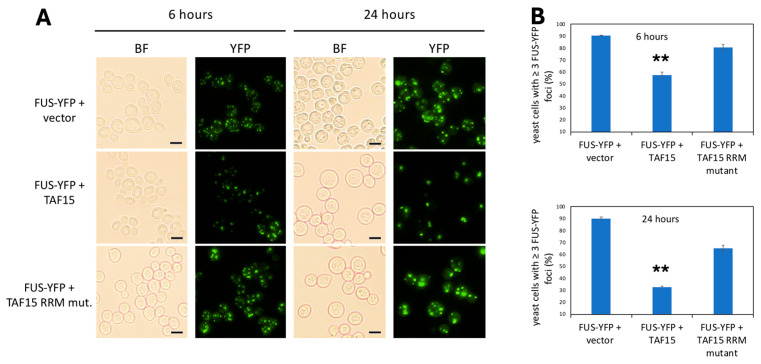
**TAF15 decreases number of FUS foci.** (**A**) Yeast transformed with a FUS-YFP expression plasmid and an empty vector, wild-type TAF15 or TAF15 RRM mutant plasmid were grown in liquid media containing 2% galactose to induce FUS-YFP and TAF15 expression. Live yeast cells were observed at 40× magnification in the brightfield and YFP channels at 6 and 24 h of growth. The same exposure time and microscope settings were used to take pictures for each strain, and representative images from three independent experiments were shown. Scale bar—5 µm. (**B**) The percentage of yeast cells with ≥3 foci were quantitated at 6 and 24 h (*n* = 500, average from three independent experiments, ** *p* < 0.01).

To determine whether TAF15 rescues FUS toxicity by reducing FUS aggregation, yeast strains expressing FUS-YFP and transformed with an empty vector, wild-type (WT) TAF15, or an RRM-domain mutant of TAF15 were grown in liquid culture. FUS aggregation was assessed at 6 and 24 h. At 6 h, the overall percentage of cells containing FUS foci did not differ significantly among the groups (Figure 3). However, the aggregation pattern was visibly altered in cells expressing WT TAF15. Specifically, these cells exhibited fewer FUS foci per cell compared to cells expressing the empty vector or the TAF15 RRM mutant (Figure 3A).

To quantify this observation, we calculated the percentage of cells containing three or more FUS-YFP foci at both time points. At 6 h, 89% of cells expressing the empty vector and 81% of cells expressing the TAF15 RRM mutant contained three or more foci, whereas only 60% of cells expressing WT TAF15 reached this threshold. This difference became more pronounced at 24 h: 90% of cells without TAF15 contained three or more foci, compared to only 29% in cells expressing WT TAF15 (Figure 3B). In addition to reducing foci number, WT TAF15 also altered aggregate morphology at 24 h. Cells expressing WT TAF15 displayed fewer small, spherical foci and instead often formed one to two larger, less spherical aggregates (Figure 3A).

### 3.4. FUS and TAF15 Induce and Localize to P-Bodies

Stress granules and p-bodies are conserved cytoplasmic ribonucleoprotein (RNP) granules that assemble via liquid–liquid phase separation in response to specific stress conditions [47,48]. Stress granules contain translation initiation factors, small ribosomal subunits and RBPs and are thought to play a role in storage of mRNA that can be translated following resolution of the inducing stress. P-bodies function in the storage and degradation of mRNA, and contain proteins involved in decapping and 5′-3′ mRNA decay, as well as nonsense-mediated decay [49,50]. FUS inclusions have been shown to colocalize with the stress granule markers PABP-1 and eIF4G in postmortem brain and spinal cord tissue from ALS patients with mutations in FUS [51]. Endogenous FUS localizes to stress granules following sorbitol stress, and ALS-linked mutant FUS localizes to stress granules under a variety of stress conditions [52,53,54]. Many ALS-linked mutations occur in proteins that affect stress granule dynamics or in proteins that localize to stress granules, leading to the hypothesis that the dysregulation of stress granules underlies ALS pathology [55,56]. TAF15 was reported to form cytoplasmic foci in yeast, but whether these foci colocalize with stress granules or p-bodies has not been studied [12].

**Figure 4:**
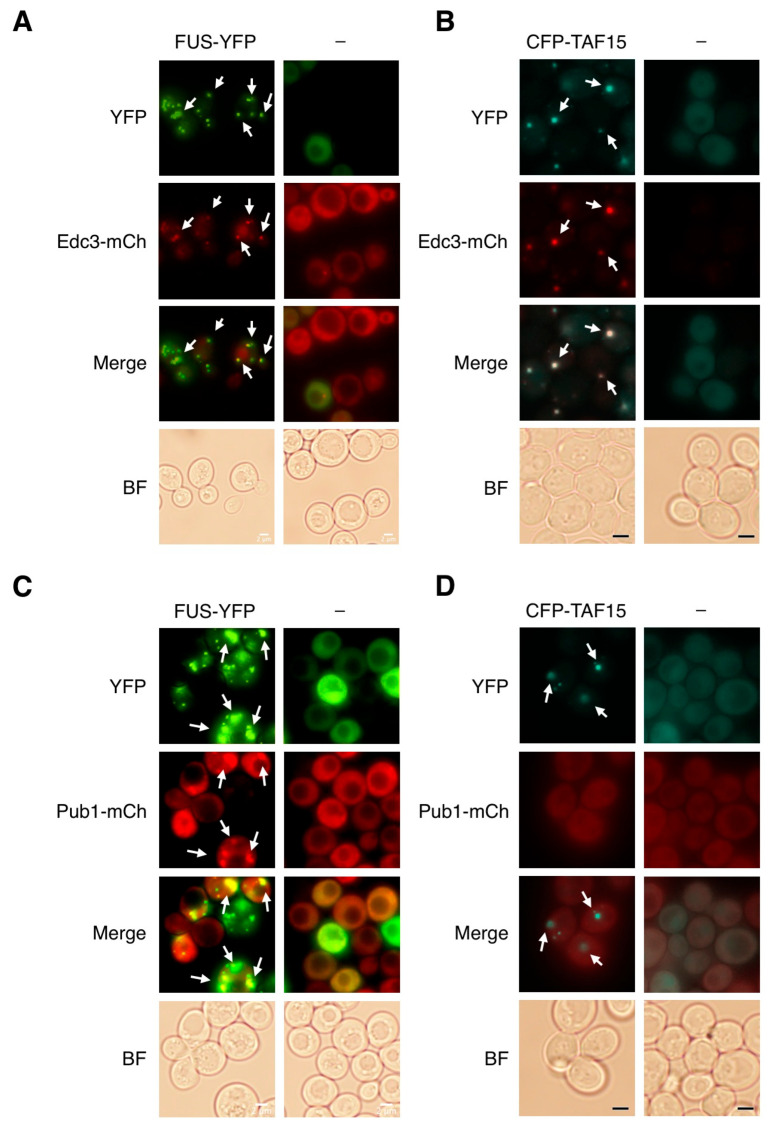
**TAF15 induces and localizes to p-bodies.** (**A**) Yeast expressing the p-body marker (Edc3-mCherry) were transformed with YFP (-) and FUS-YFP plasmids and induced with 2% galactose for 6 h followed by microscopy. (**B**) Yeast expressing the p-body marker (Edc3-mCherry) were transformed with ECFP (-) and ECFP-TAF15 plasmids and induced with 2% galactose for 6 h followed by microscopy. (**C**) Yeast expressing the stress granule marker (Pub1-mCherry) were transformed with YFP (-) and FUS-YFP plasmids and induced with 2% galactose for 6 h followed by microscopy. (**D**) Yeast expressing the stress granule marker (Pub1-mCherry) were transformed with ECFP (-) and ECFP-TAF15 plasmids and induced with 2% galactose for 6 h followed by microscopy. Images are representatives of three independent experiments. Scale bar—2 μm.

To test whether FUS and TAF15 localize to either or both of these RNP structures, we individually expressed FUS-YFP and ECFP-TAF15 in yeast containing mCherry-tagged p-body marker (Edc3) and stress granule marker (Pub1). The overexpression of FUS and TAF15 both induce the formation of p-bodies and both proteins colocalize with p-bodies (Figure 4A,B). In contrast, FUS, but not TAF15, induces the formation of stress granules (Figure 4C,D). FUS inclusions colocalize with Pub1-labeled stress granule structures (Figure 4C), while Pub1 remains diffusely localized in the cytosol, when TAF15 is expressed alone (Figure 4D). Interestingly, FUS aggregates almost completely colocalize with p-bodies but only partially with stress granules, suggesting that the observed FUS foci are largely accounted by p-bodies rather than stress granules.

### 3.5. TAF15 Enhances P-Body Formation When Co-Expressed with FUS

**Figure 5:**
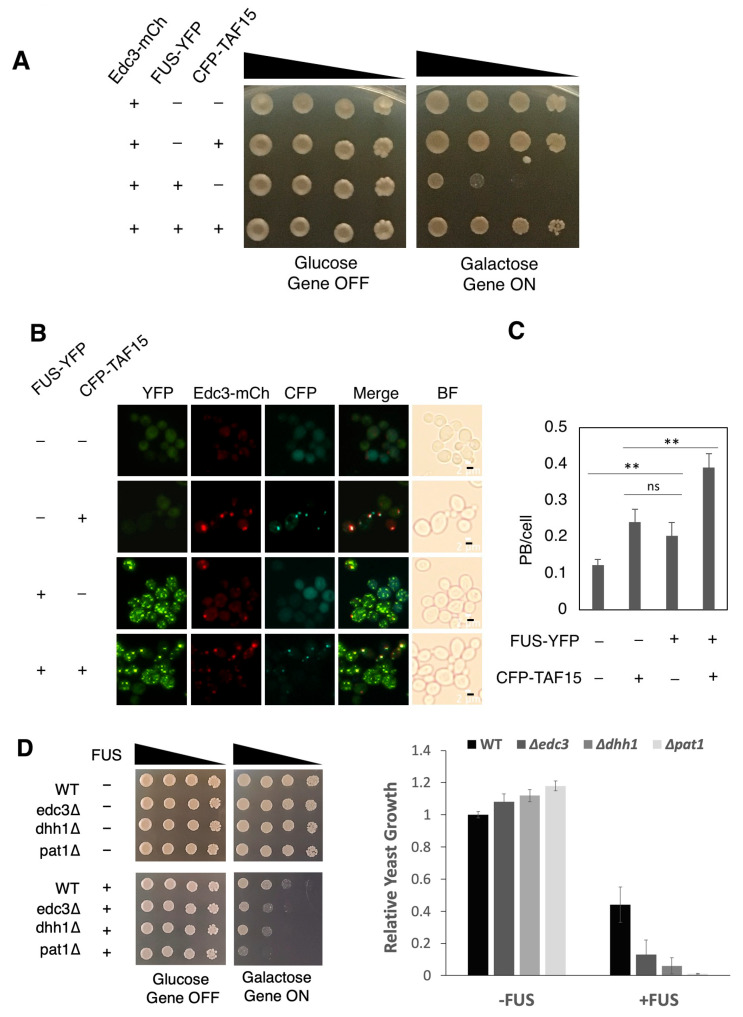
**TAF15 increases the induction of p-bodies when co-expressed with FUS**. (**A**) Serial dilution growth assay was performed using yeast strains expressing the p-body marker (Edc3-mCherry) and transformed with FUS-YFP, ECFP-TAF15, or both. Wild-type yeast strain transformed with a YFP and ECFP plasmid was used as a control strain showing no toxicity. (**B**) Yeast strain expressing the p-body marker (Edc3-mCherry) was transformed with FUS-YFP, ECFP-TAF15, or both. The same yeast strain transformed with ECFP and YFP were used as controls without the expression of FUS or TAF15. All yeast strains were induced with 2% galactose for 6 h. Live yeast cells were observed at 100× magnification in the YFP, ECFP, TxRed and BF channels. The same exposure time and microscope settings were used to take pictures for each strain. Scale bar—2 μm. (**C**) Quantification of the number of p-bodies per cell after 6 h of galactose induction. Data generated from three biological replicates. ns not statistically significant; ** *p* < 0.01. (**D**) Serial dilution growth assay was performed using yeast strains with deletions in genes involved in p-body formation EDC3 (edc3Δ), DHH1 (dhh1Δ), and PAT1 (pat1Δ) transformed with empty vector pRS413Gal1YFP (-) and FUS expression construct pRS413FUS-YFP (+). Isogenic wild-type strain (WT) transformed with the empty vector (-) and FUS expression constructs (+) were used as controls. Images are representatives of three independent experiments. Data shown on the right were generated from three biological replicates using Image J to quantitate the colonies formed on the second dilution when the gens were turn on (galactose). ** *p* < 0.01.

Considering that both RNA-binding and physical interaction with FUS are required for TAF15 to suppress FUS toxicity, we reasoned that p-body formation may be involved in the observed rescuing effect. We therefore examined the formation of p-bodies in yeast strains expressing both FUS and TAF15. We transformed yeast with both ECFP-tagged TAF15 and YFP-tagged FUS. Similar to the non-tagged TAF15 protein, ECFP-tagged TAF15 rescues FUS toxicity (Figure 5A). Similar to what we found in Figure 4A,C, the individual expression of FUS and TAF15 induced the formation of p-bodies and both proteins co-localize with p-bodies (Figure 5B). When FUS and TAF15 were co-expressed, a greater number of p-bodies (50% increase) were detected compared to when there was individual expression of FUS and TAF15 (Figure 5B,C). These data support a possible role of additional p-bodies in the rescue of FUS toxicity by TAF15.

To further explore the potential role of additional p-bodies in rescuing cells from FUS toxicity, we considered p-bodies associated yeast genes. Edc3, Dhh1 and Pat1 are important regulators of p-body assembly in yeast. Deletions of each of these three genes cause deficiency in the formation of p-bodies [57,58,59]. To test the role of p-bodies in FUS toxicity, we expressed FUS in three mutant yeast strains with *EDC3*, *DHH1*, or *PAT1* deleted. The deletion of each of the three genes alone does not elicit any growth defect. In contrast, FUS toxicity is markedly enhanced in these deletion mutants. These data support the idea that formation of p-bodies is critical in mitigating cellular toxicity induced by FUS (Figure 5D).

### 3.6. TAF15 RRM and ΔN Mutants Induce and Localize to P-Bodies

**Figure 6:**
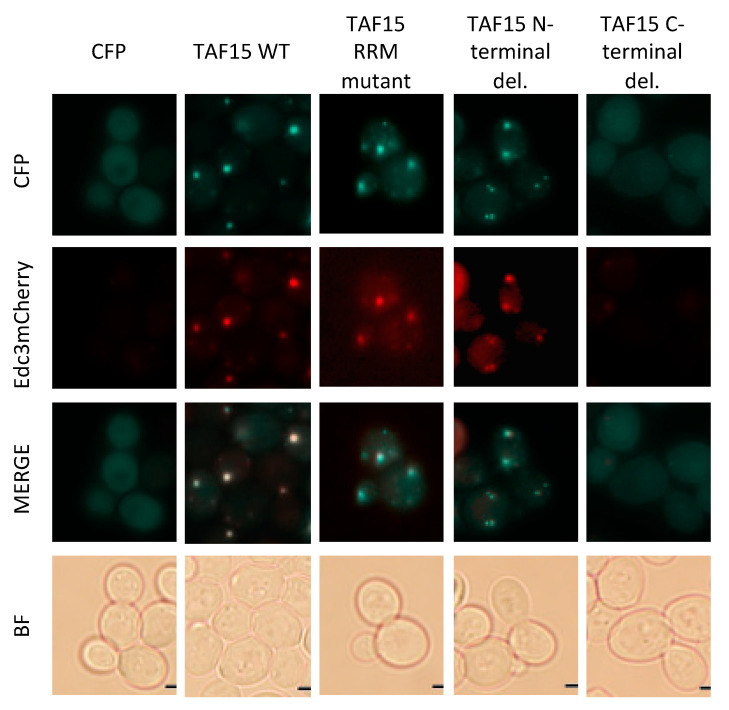
**TAF15 RRM mutant and ΔN mutant induces and colocalize with P-bodies**. Expression of WT and mutant CFP-TAF15 were induced for 6 h with 2% galactose in yeast containing the p-body marker Edc3-mCherry. Live yeast cells were observed at 100× magnification in the CFP, TxRed and BF channels. The same exposure time and microscope settings were used to take pictures for each strain. Images shown are representatives from three independent experiments, scale bar—2 µm.

To determine whether p-body induction and colocalization by TAF15 are required for alleviating FUS toxicity, we examined TAF15 mutants that fail to rescue FUS-mediated toxicity and assessed their ability to induce and/or localize to p-bodies. P-bodies were largely absent in yeast expressing CFP alone. As expected, the expression of CFP-TAF15 induced p-body formation and colocalized with p-bodies. Notably, both the TAF15 RRM mutant and the N-terminal deletion (ΔN) mutant also induced p-body formation and colocalized with p-bodies (Figure 6). In contrast, the C-terminal deletion mutant displayed diffuse localization throughout the cell and did not colocalize with p-bodies (Figure 6). These results indicate that the localization of TAF15 to p-bodies, by itself, is not sufficient to rescue FUS toxicity, as the RRM and ΔN mutants retain p-body association but fail to confer protection.

## 4. Discussion

Mutations in a number of RBPs have been linked to neurodegenerative diseases such as ALS and FTD, and accumulating evidence suggests that the dysregulation of RNA metabolic processes is an underlying mechanism contributing to disease pathogenesis [60,61,62,63]. Evidence from in vitro and in vivo models of FUS pathology point to cytoplasmic mislocalization and accumulation into stress granules as drivers of FUS toxicity through a variety of proposed mechanisms. These mechanisms include the sequestration of essential mRNA and RNA-binding proteins, the disruption in mRNP dynamics and the dysregulation of NMD, to name a few [15,16,53,64,65,66]. Previous yeast genome-wide overexpression and deletion screens using a yeast model of FUS toxicity identified RBPs and proteins involved in RNA metabolism as modifiers of FUS toxicity [15,16]. In a newly developed genome-scale genetic screen, we identified human genes that strongly suppress FUS toxicity [26]. The identified suppressors are enriched in genes encoding RBPs. Among them is TAF15, which belongs to the same FET protein family as FUS.

TAF15 has no yeast homolog, making it a unique suppressor that could not be identified by a homologous relationship to yeast suppressor genes. TAF15 does contain conserved protein domains found in yeast proteins such as its prion-like domain, RNA recognition motif, and RGG motif, and may target RNA in yeast cells. Notably, while the expression of FUS causes robust toxicity in yeast, TAF15 expression is well-tolerated and does not lead to overt toxicity. A yeast screen of 133 human RRM-containing proteins identified 38 RNA-binding proteins that cause toxicity to yeast, with only seven that cause a similar degree of toxicity as FUS [12]. Indeed, not all RBPs cause toxicity in yeast when overexpressed, and further exploration of the differences in properties of RBPs accounting for their toxicity would be relevant for understanding neurodegeneration in the context of the disruption of cellular functions linked to specific RBPs.

FUS toxicity is dependent on RNA binding. Given the similarity in RNA binding profiles between FUS and TAF15, we speculate that TAF15 may be competing with FUS for binding RNA targets, thus preventing FUS from mediating RNA binding-dependent toxicity. When both FUS and TAF15 are expressed at normal levels, RNA homeostasis is preserved. When such balance is disrupted by the overexpression or mutations of FUS, a higher level of TAF15 is required to restore RNA homeostasis. Interestingly, the mutation of conserved phenylalanine residues in the RRM of TAF15, which are important for RNA binding, eliminate the ability of TAF15 to rescue FUS toxicity (Figure 2B). This finding supports an essential role of the RRM domain in the suppression mechanism. It is tempting to speculate that TAF15 restores cell growth in the presence of toxic levels of FUS by competing with FUS to bind to the same RNA targets. While RNA targeted by FUS may lead to cellular toxicity, RNA bound to TAF15 would not. In this regard, it is also worth noting that EWSR1 had no effect on FUS toxicity. In fact, there is greater similarity between the RRMs of FUS and TAF15 as compared to the RRM of EWSR1. EWSR1 with a less similar RRM may be less effective in competing for the RNA targets of FUS.

In addition to RNA binding, our data suggest that a protein–protein interaction between FUS and TAF15 is required for the rescue. However, we acknowledge that this conclusion is based solely on yeast two-hybrid (Y2H) assays and therefore should be interpreted with caution given the limitations of this approach. Deletion of the C-terminal region of TAF15, which is critical for its interaction with FUS, eliminates its ability to rescue FUS toxicity (Figure 2D–F). Levels of FUS protein, however, are unchanged in the presence of TAF15 (Figure 1B). Moreover, FUS remains localized to cytoplasmic foci in the presence of TAF15 (Figure 1D), indicating that TAF15 bypasses FUS toxicity without directly acting on the FUS protein. Given that deficiency of FUS in animals leads to a variety of issues including perinatal lethality, genomic instability and hippocampal vacuolation [67,68] and that depletion of Cabeza, the drosophila homolog of FUS, results in locomotor defects [69,70,71], bypassing FUS toxicity without affecting the protein itself may be therapeutically beneficial.

The rescue by TAF15 is specific to FUS as TAF15 has no effect on the toxicity of other neurodegenerative disease-associated proteins. It is not surprising that TAF15 had no effect on the toxicity of HTT103Q and α-syn. Yeast models of protein misfolding diseases can differ markedly in their biochemical properties and cellular effects. For example, the toxicity and aggregation of Htt103Q can be alleviated by deleting either the heat shock protein HSP104 or the yeast prion protein RNQ1. However, these same genetic deletions do not suppress the cytoplasmic aggregation or toxicity of FUS in yeast [16]. In addition, Htt103Q forms SDS-insoluble aggregates that are retained by a 0.2 µm cellulose acetate filter, whereas FUS aggregates are not similarly retained and can pass through [16]. The identified functions of α-syn differ widely from those identified for FUS [28,72]. The structural and functional differences between the two proteins could be the reason why TAF15 has no effect on α-syn. TDP-43 and FUS, however, are both RBPs containing prion-like domains as well as other shared domains. Like FUS, TDP-43 has been shown to colocalize with stress granules and p-bodies in yeast [36,72]; it is interesting that TAF15 had no effect on TDP-43 toxicity. In this regard, it is notable that using the same Y2H assay, we could detect the interaction of TAF15 with FUS but not with TDP-43. The lack of physical interaction may account for the lack of rescue.

Pull-down experiments with FUS and TAF15 have previously shown that incubation of the protein complex with DNase and/or RNase did not affect the binding between FUS and TAF15 [44], suggesting that RNA molecules bound to FUS and TAF15 are not necessary for maintaining protein–protein interaction between the two proteins. Consistent with this finding, no difference was observed in the interaction strength with FUS between the RRM mutant and wild-type TAF15 (Figure 2F).

Considering our observations that FUS localizes to both p-bodies and stress granules, while TAF15 exclusively localizes in p-bodies (Figure 4), we speculate that formation of p-bodies may be protective. Consistent with this idea, the overexpression of the yeast core p-bodies proteins Sbp1 and Edc3 were both found to suppress FUS toxicity through genome-wide screens [15,16]. To test if the induction of p-bodies may be protective, we quantified the number of p-bodies in yeast strains expressing FUS and TAF15 individually and together. Consistent with our hypothesis, yeast co-expressing FUS and TAF15 contained more p-bodies per cell as compared to yeast expressing each protein individually (Figure 5B,C). It is tempting to speculate that, in the presence of TAF15, FUS might be targeted from toxic stress granules to protective p-bodies. This hypothesis is supported by our finding that FUS toxicity is markedly enhanced in yeast deletion strains defective in p-body formation (Figure 5D). It is worth noting that, unlike mammalian cells where stress granules are the primary cytoplasmic RNP granules, yeast cells contain both stress granules and p-bodies, which are closely related yet distinct structures. This difference in cellular context may contribute to the observed divergence in TAF15 localization.

## Data Availability

The original contributions presented in this study are included in the article/Supplementary Material. Further inquiries can be directed to the corresponding author(s).

## Supplementary Materials

The following supporting information can be downloaded at: https://www.mdpi.com/article/10.3390/jof12050341/s1, Figure S1: TAF15 does not change protein levels of FUS. Protein expression was examined in 1XFUS model transformed with an empty vector or TAF15 expression plasmid. Yeast containing two empty vectors was used as a control strain without FUS or TAF15 expression. Protein expression was induced with 2% galactose and proteins were harvested from the yeast at 3 and 22 h as indicated. Western blot was performed using an antibody against FUS and the loading control protein PGK1. The cropped images of Western blots were shown here for the purpose of clarity. The full-length blots are presented in Supplemental Figure S3; Figure S2: FUS-YFP on a centromere plasmid shows comparable toxicity as non-tagged FUS integrated into genome. Serial dilution growth assay was performed using wild-type yeast transformed with the vector control pRS415Gal1ccdB (-), non-tagged FUS pRS303Gal1FUS integrated into to the genome (FUS), and C-terminal YFP-tagged FUS pRS413Gal1FUS-YFP (FUS-YFP). The picture shown was taken after 2 days growth at 30 °C incubation, and is a representative of three independent experiments; Figure S3: Uncropped original images of the Western blots.
