# Supplementary material for: RNA-Binding Protein TAF15 Suppresses Toxicity in a Yeast Model of FUS Proteinopathy
Source: J Fungi (Basel). 2026 May 6;12(5):341. doi: 10.3390/jof12050341 (PMC13208891; doi:10.3390/jof12050341)
Supplement: Supplementary file 1 [file jof-12-00341-s001.zip › jof-4239543-supplemental figures.pdf]

Article

RNA-Binding Protein TAF15 Suppresses Toxicity in a Yeast Model of FUS Proteinopathy

Elliott Hayden [1], Aicha Kebe [1], Shuzhen Chen [1], Abagail Chumley [1], Chenyi Xia [2], Widad El-Zein [1], Quan Zhong [1,*] and Shulin Ju [1,*]

[1] Department of Biological Sciences, Wright State University, Dayton, OH 45435, USA; hayden.24@wright.edu (E.H.); kebe.3@wright.edu (A.K.); chens@marshall.edu (S.C.); chumleyabby@gmail.com (A.C.); el-zein.2@wright.edu (W.E.)
[2] School of Basic Medicine, Shanghai University of Traditional Medicine, Shanghai 201203, China; chenyixia@shutcm.edu.cn
* Correspondence: quan.zhong@wright.edu (Q.Z.); shulin.ju@wright.edu (S.J.)

Supplemental: Figures

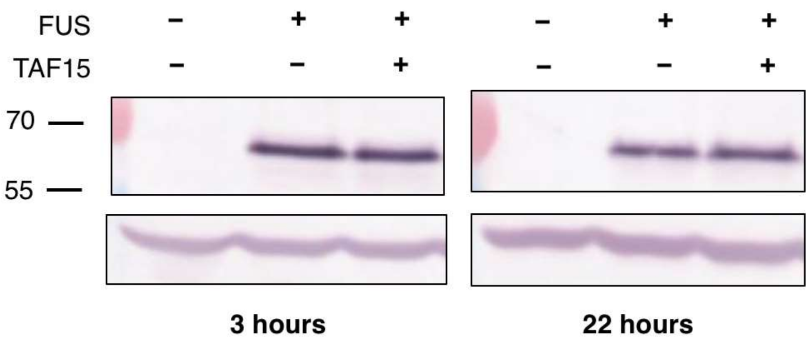

Figure S1. TAF15 does not change protein levels of FUS. Protein expression was examined in 1XFUS model transformed with an empty vector or TAF15 expression plasmid. Yeast containing two empty vectors was used as a control strain without FUS or TAF15 expression. Protein expression was induced with 2% galactose and proteins were harvested from the yeast at 3 and 22 hours as indicated. Western blot was performed using an antibody against FUS and the loading control protein PGK1.

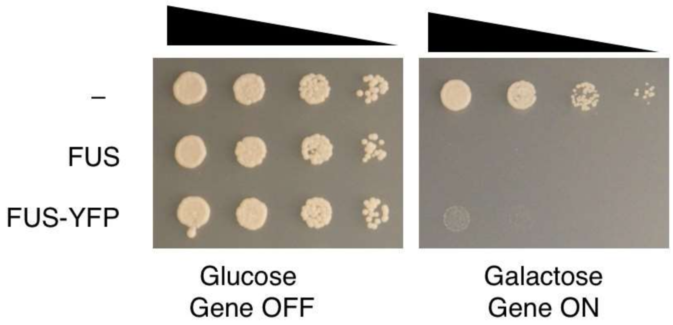

Figure S2. FUS-YFP on a centromere plasmid shows comparable toxicity as non-tagged FUS integrated into genome. Serial dilution growth assay was performed using wild-type yeast transformed with the vector control pRS415Gal1ccdB (-), non-tagged FUS pRS303Gal1FUS integrated into to the genome (FUS), and C-terminal YFP tagged FUS pRS413Gal1FUS-YFP (FUS-YFP). The picture shown was taken after 2 days growth at 30°C, and is a representative of three independent experiments.

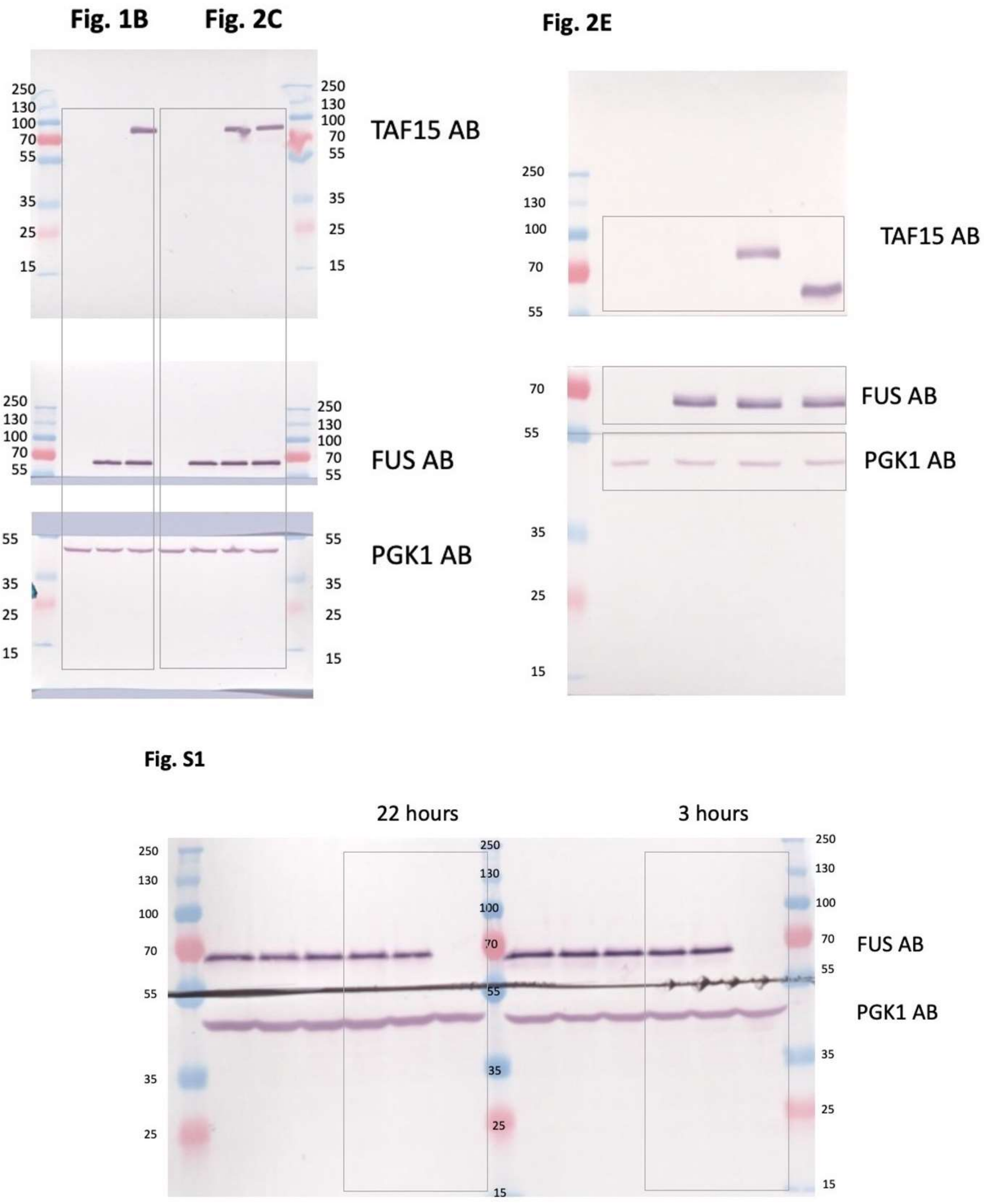

Figure S3. Uncropped original images of the Western blots.
